# Lesser known indigenous vegetables as potential natural egg colourant in laying chickens

**DOI:** 10.1186/2055-0391-56-18

**Published:** 2014-09-24

**Authors:** Bolu Steven Abiodun, Aderibigbe Simeon Adedeji, Elegbeleye Abiodun

**Affiliations:** Department of Animal Production, University of Ilorin, Ilorin, Nigeria

**Keywords:** Canthaxanthin, Egg yolk colorant, Waterleaf, Red pepper, Baobab leaf

## Abstract

**Background:**

A six-week study involving two hundred and fifty (250) Harco Black layer birds at point of lay was conducted to investigate the effects of potential natural colorant on performance and Egg quality traits. The birds were assigned to five (5) dietary treatments, each containing supplements either of control, Baobab Leaf (BL), Waterleaf (WL), Red Pepper (RP), Canthaxanthin (CTX) at 40 g/kg feed and 50 mg/kg feed of natural and commercial colorants, respectively.

**Results:**

Performance records shows that there was no significant (p > 0.05) difference in feed intake across the supplements of Red pepper, Water leaf, Canthaxanthin and control diet, however, birds fed Baobab leaf treatment had a significantly lower (p < 0.05) feed intake value (94.07 g) when compared with other treatments. Body weight gain and Hen Day Production were not significant influenced (p > 0.05) by the dietary treatments, although laying hens fed Baobab leaf supplement had lowest mean HDP of 48.80%, while birds fed Red pepper and Water leaf supplement had an average value of 52.79%. There was no significant effect (p > 0.05) of colorants on egg external traits, compared with the control; birds fed Canthaxanthin treatment had higher mean egg weight (51.79 g), egg length (4.55 g), egg breadth (3.29 g); Red pepper treatment had highest mean shell thickness (0.29 g), however these differences were not significant (p > 0.05). Yolk height, Albumen height, Yolk index, and Haugh unit were not significantly affected (p > 0.05) across treatments. Yolk width was lowest (p < 0.05) in Baobab leaf treatment (2.54 cm); Red pepper, Water leaf and Canthaxanthin (2.89 cm, 2.62 cm and 2.89 cm respectively) were not significantly (p > 0.05) different from the control (2.73 cm). Yolk colour score was significantly highest (p < 0.05) in Red pepper treatment (7.50); Water leaf, Baobab leaf and Canthaxanthin ranged between 2.25- 3.31 on the DSM yolk colour fan, Control treatment had the lowest yolk colour score (p < 0.05) of 1.31.

**Conclusion:**

The study showed Red pepper as a worthy alternative to commercial yolk colorant. Water leaf and baobab are not good substitutes for canthaxanthin as a yolk colourant.

## Background

Eggs are used in various food industries in the manufacture of confectionery cosmetics, production of vaccine as reported by [1]. Egg yolk colour is a major concern to consumers as it affects their purchasing behavior [2]. The colour of the egg yolk is considered to be one of the important factors for egg consumption. Consumers select eggs based on the egg yolk color and other egg qualities. However, laying birds cannot produce coloring pigments that is known to enhance yolk coloration to meet the demand of consumer for the desired colored egg yolk and egg quality and thus, depends directly on dietary supply for egg yolk coloration [3]. Indeed, the perception of the intensity of the yolk colour depends directly on the quantity of feed consumed, the transfer efficacy and chemical composition of the carotenoid source [4]. The efficiency of pigment source depends on the digestibility, transfer, metabolism, and deposition of carotenoids in target tissue and upon their colour hue [5]. The most effective carotenoids in poultry diets are the synthetic forms which have been manufactured to ensure high transfer efficiency and colouring capacity (apoester: 50-60%; canthaxanthin 30-50%) [6]. The main vegetable sources of carotenoids are corn, corn gluten, lucerne, lucerne concentrates and flower (marigolds, tagetes) and plant (paprika) extracts. Synthetic oxy-carotenoids which corresponded to natural carotenoids (canthaxanthin, citranaxathin) have been chosen in the laying hen rations for their colouring effectiveness, (which is two to three times more efficient as a yolk colorant than carotenoids of vegetable origin), and for their high stability (due to encapsulation against oxidation and degradation) [7]. These products are, however, banned in organic production, which rely mainly on yellower plant sources, explaining the paler yolk colouration found in this system of poultry production. Canthaxanthin has also been reported as a potential skin and eye irritant, and it is an expensive source of yolk colorant [8]. In practice, satisfactory colour of table eggs can be obtained with small amounts of yellow xanthophylls (15–25 mg/kg) combined with 1–2 mg/kg red carotenoids.

The attractive red color of peppers, yellow color of ripe pawpaw and the green pigment of vegetables (*Talinium triangulare*, *Telfairia occidentalis, Adansonia digitata, etc.*) is due to their various carotenoid pigment. These carotenoids include; capsanthin, capsorubin and cryptocapsin [8]. Maize (lutein), Red pepper (capsanthin and capsorubin), Fluted pumpkin*,* Pawpaw (cryptoxanthin), Sweet potato leaf and Marigold (lutein) contain colorants that can be used as feed supplements in diet of laying birds and are known to improve egg quality and yolk pigmentation*.*

Vegetable sources provide mainly yellow carotenoids. Paprika oleoresin is prepared from the dried fruit of *red pepper* by a similar process to *tagetes* involving dehydration, solvent extraction, saponification and stabilization are useful carotenoids for egg yolk coloration. Its effectiveness ratios relative to canthaxanthin varies from 3 to 5 for yolk deposition yield and 1 to 4 for pigmentation ability. Extract from algae containing Asthaxanthin (3,3′-dihydroxycanthaxanthin) has been used in combination with yellow xanthophylls for egg yolk coloration, but the rate of deposition of carotenoids in the egg yolk is lower than that of canthaxanthin [9].

This study focused on the use of lesser known indigenous vegetables as natural pigmenting plants; Red pepper (*Capsicum annuum*), Water leaf (*Talinum triangulare*) and Baobab leaf (*Adansonia digitata*), with the aim of improving egg quality traits through egg yolk coloration.

## Method

### Experimental animals and management

The experiment was carried out at the Teaching and Research farm, Faculty of Agriculture, University of Ilorin. Two hundred and Fifty (250) twenty-week old Black Harco layers were used for the experiment that lasted six weeks. The laying hens were housed in a 2-tiers battery cage system. Diet was formulated to meet NRC [10] requirements for energy and protein of laying birds. Each natural plant colorants (Red Pepper, Baobab Leaf and Waterleaf) and a commercial yolk colorant (Canthaxanthin) were incorporated into the diet at separate rates of 40 g/kg and 50 mg/kg of feed, respectively. Other routine management practices such as vaccination, medication and proper hygiene recommended by animal science regulations in Nigeria were complied with.

The leaves of *Adansonia digitata* (Baobab) (BL)*, Capsicum annuum* (Red pepper)(RP) and *Talinium triangulare* (Water leaf) (WL) were collected, destalked and washed. The leaves were air dried for four days to reduce the moisture content and achieve a constant dry weight while retaining their colours. After drying, the leaves were pulverized into fine powder using an electric blender (Moulinex Philips). A known commercial egg yolk colorant (Canthaxanthin, CTX) was procured from a commercial feedmill in Ilorin, Nigeria. Experimental Birds were randomly assigned to five treatments using the Completely Randomized design (CRD) comprising fifty (50) birds per treatments. Each treatment was replicated in five units of ten [10]. The treatments were allocated as such; Diet A containing *Capsicum annuum* as colorant at 40 g/kg of feed, Diet B containing *Talinium triangulare* as colorant at 40 g/kg of feed, Diet C containing *Adansonia digitata* as colorant at 40 g/kg of feed, Diet D containing Canthaxanthin at 50 mg/kg of feed, and Diet E (Control) diet with no yolk colorant (Table 1).

**Table 1:** **Composition of experimental diet**

Feed ingredients	Percentage %
White maize	45.0
Corn bran	10.0
Brewer’s dried grain	10.0
Wheat offal	4.50
Fish meal (72%)	1.44
Soyabean meal	20.0
Bone meal	0.26
Oyster shell	8.00
Vitamin/mineral premix	0.25
Lysine	0.10
Methionine	0.15
Salt	0.30
Total	100

Nutrient composition according to (8).

### Data collection

Data were collected for performance and egg quality assessment. Average Feed Intake (AFI) was recorded daily, while Body weight gain (BWG) was recorded weekly as follows:


F_1_ = left over feed at the end of the day (g)

F_0_ = feed offered in the morning (g)


W_1_ = weight change at the end of the week

W_0_ = weight at the beginning of the week

Hen-Day Production (HDP) was calculated using the formula:


At the end of each week, fifty eggs per treatment were selected for analysis. Parameters measured include; Egg Weight (EW), Egg Length (EL), Egg Breadth (EB), Yolk Height (YH), Yolk Width (YW), Albumen Height (AH), Shell Thickness (ST), Yolk Colour (YC), Yolk Index (YI)and Haugh Unit (HU).

EW was collected by cleaning the eggs to remove impurities and weighing them using a sensitive electronic scale (Metler). ST was determined using a pair of micrometer screw gauge calibrated in millimetres. The accuracy of ST was ensured by measuring shell sample as one egg at the broad end, middle portion, and the narrow end all referred to as the thin, medium and thick, respectively. The average of these three parts measured was taken as the ST. YI was determined by relating the ratio of YH in millimetres to the YW measured in millimetres. The YH and YW were measured using a Spherometer and Venier calliper, respectively. YW was also taken as the maximum cross sectional diameter of the yolk which is the width at maximum point, usually across the centre of the yolk. The YC of collected eggs were determined using DSM yolk colour fan.

Albumen was separated from the yolk and carefully placed on a flat surface. The AH was measured using a Spherometer calibrated in millimetres. HU was measured relating the albumen height and the egg weight from the formula:


H.U = Haugh unit

H = Observed albumen height.

W = Observed weight of egg in grams.

### Statistical analysis

Data obtained from the experiment were subjected to analysis of variance for completely randomized experimental design using the SPSS package, according to [11]. Significant means were separated using the Duncan’s Multiple Range Test [12].

## Results and discussion

### Effects of potential natural colorant on bird’s performance

Birds differed significantly (P < 0.05) in AFI across the various treatments (Table 2). Diet supplemented with BL recorded a significantly lower (P < 0.05) mean intake value (94.07 g) when compared with other treatments. This could be due to the anti-nutritional agent present in baobab leaf. It has been reported [13] that high tannin content of BL prevent its use as a potential feed ingredient in poultry diets. However, BL has been used as a source of pigments in diets for laying birds where yolk colour was observed to have increased at 1% or 2% BL, with no adverse effects on feed intake, egg production and quality. Canthaxanthin has been reported to have no influence on AFI [6].

**Table 2:** **The relative effects of the potential natural colorant on Bird’s performance**

Parameter	***Capsicum annuum***	***Talinium triangulare***	***Adansonia digitata***	Canthaxanthin	Control	±SEM
BWG (g)	950 ± 0.06^ab^	110 ± 0.03^b^	470 ± 0.26^a^	630 ± 0.15^ab^	750 ± 0.45^ab^	0.03
AFI (g/bird/d)	101.33 ± 3.32^b^	103.16 ± 3.13^b^	94.07 ± 1.37^a^	103.56 ± 1.39^b^	104.36 ± 2.24^b^	0.54
HDP	52.97 ± 17.99	52.97 ± 19.09	48.80 ± 16.22	58.34 ± 18.91	58.60 ± 20.08	4.14

*a, b, ab,* Means across same row carrying different superscripts are significant (p < 0.05).

HDP increased comparatively over the period of experiment. Laying hens fed with the control diet had the highest mean value (58.60%) while the diet supplemented with BL had the lowest value (48.8%). RP supplemented diet did not influence (P > 0.05) the performance parameters measured viz: Body weight gain, feed intake, Hen-day production. The observation on HDP disagreed with the report of [6] that CTX increased egg production. CTX has been reported to have no effects on HDP [14].

There was no dietary effect of treatment (P > 0.05) on egg external quality traits (Table 3). Canthaxanthin and other dietary supplements had no significant effects on egg external quality traits such as; EW, EB, EL, EI and ST; this is consistent with the previous findings [15]. This study showed a shell thickness range of 0.27 mm-0.29 mm which is within normal range for poultry. The Egg ST is an important trait for hatchability and handling [16].

**Table 3:** **Effects of potential natural colorant on egg external quality traits**

Parameter	***Capsicum annuum***	***Talinium triangulare***	***Adansonia digitata***	Canthaxanthin	Control	±SEM
Egg weight	48.27 ± 1.20	47.56 ± 6.68	46.87 ± 5.20	51.79 ± 2.10	49.84 ± 2.34	0.93
Egg length	4.35 ± 0.06	4.24 ± 0.60	4.20 ± 0.46	4.55 ± 0.16	4.37 ± 0.13	0.08
Egg breadth	3.23 ± 0.09	3.08 ± 0.51	3.02 ± 0.39	3.29 ± 0.06	3.16 ± 0.30	0.07
Egg index	1.35 ± 0.48	1.38 ± 0.63	1.39 ± 0.03	1.38 ± 0.06	1.38 ± 0.05	0.01
Shell thickness	0.29 ± 0.18	0.27 ± 0.18	0.27 ± 0.44	0.28 ± 0.02	0.27 ± 0.03	0.02

Means across same row carrying different superscripts are significant (p < 0.05).

The various dietary treatments had no significant effect (P > 0.05) on egg internal qualities such as: AH, YH, YI and HU. This is also consistent with earlier reports [10].

There was however, a significant effect (P < 0.05) of dietary treatment on YC (Table 4). Laying hens fed Red pepper supplemented diet had significantly higher (P < 0.05) YC (colour index of 7.50) compared with the other treatments. However, diets supplemented with WL, BL (*Adansonia digitata*) and CTX had similar (P > 0.05) values for YC. CTX was reported (6) to enhance egg yolk pigmentation. The observed low YC score for CTX in this study may be related to reduced potency owing to post-importation storage. The control diet had significantly lowest YC score which further buttress the fact that yolk pigmentation is influenced mostly by diets given to the birds [17]. In this study, RP is the best natural source of potential egg colourant, this is consistent with the report of [18]. That canthaxanthin had similar score with other lesser known indigenous vegetables shows that these vegetables can be harnessed into layer diets for egg yolk colour pigmentation. BL and WL are potential good sources of xanthophylls, known to enhance yolk pigmentation. Natural xanthophylls are well absorbed by hen intestinal cells [19] and it is transferred into the yolk after being released into the circulatory system [20].

**Table 4:** **Effects of natural colorant on internal egg quality traits**

Parameter	***Capsicum annuum***	***Talinium triangulare***	***Adansonia digitata***	Canthaxanthin	Control	±SEM
Yolk height (mm)	14.83 ± 0.05	13.30 ± 2.07	13.75 ± 1.58	14.53 ± 0.52	14.62 ± 0.42	0.27
Yolk index	5.15 ± 0.84	4.81 ± 0.76	5.19 ± 0.86	5.04 ± 0.11	5.37 ± 0.14	0.12
Yolk width (cm)	2.89 ± 0.06	2.62 ± 0.37	2.54 ± 0.25	2.89 ± 0.12	2.73 ± 0.64	0.05
Albumen height (mm)	5.49 ± 0.21	5.06 ± 1.04	5.41 ± 1.19	5.78 ± 0.43	5.54 ± 0.30	0.17
Haugh unit	77.41 ± 1.68	76.08 ± 2.22	78.21 ± 4.03	77.54 ± 2.58	76.93 ± 2.25	0.60
Yolk color	7.50 ± 1.47^c^	3.31 ± 0.83^b^	3.06 ± 0.13^b^	2.25 ± 0.20^ab^	1.31 ± 0.13^a^	0.17

*a, b, ab, c* Means across same row carrying different superscripts are significant (p < 0.05).

## Conclusion

This study shows that lesser known indigenous vegetables are potential natural egg yolk colorants. RP supplement had superior performance in this regard over canthaxanthin (imported commercial egg yolk colourant). The period of importation and post-importation storage may have reduced the potency of CTX. The use of natural colorant such as lesser known indigenous vegetables is of utmost importance to improve yolk pigmentation and performance of birds with no adverse effect as compared to artificial colourants which could be of environmental and public health concern. Powdered RP supplementation could be used as an alternative for synthetic commercial yolk colorant which is quite expensive and potentially unhealthy. The use of yellow maize based diet with powdered WL and BL supplement can also help improve yolk color and performance.
